# GSK-3 Inhibitors and Tooth Repair: An Ethical Analysis

**DOI:** 10.3389/fphar.2018.01495

**Published:** 2019-01-07

**Authors:** Sorin Hostiuc, Paula Perlea, Mihai Marinescu, Catalin Dogaroiu, Eduard Drima

**Affiliations:** ^1^ Carol Davila University of Medicine and Pharmacy, Bucharest, Romania; ^2^ University of Medicine and Pharmacy, Galaţi, Romania

**Keywords:** Tideglusib, Alzheimer’s disease, dental cavities, stem cells, non-maleficence, autonomy, dental beneficence

## Abstract

Tideglusib^®^, a GSK-3 inhibitor, was initially tested for the treatment of Alzheimer’s disease. However, a recent report has suggested its potential off-label use for the treatment of dental cavities. Even if this effect is not yet confirmed, this off-label use can have significant public/dental health consequences, mainly because of the large number of patients with cavities. The purpose of this mini-review is to perform an ethical analysis of the use of Tideglusib in dentistry. The ethical analysis identified three main areas in which ethical breaches could be significant: 1) respect for the autonomy of the patient, 2) issues raised by horizontal shifts in the translational research process, and 3) the conflict between dental beneficence and general non-maleficence. In conclusion, the use of Tideglusib in dentistry should respect the same strict ethical and regulatory criteria from clinical medicine. A translation of the potential risks should be done only after large-scale, phase-III/IV clinical trials, explicitly designed to test the usefulness of this drug in dental medicine.

## Introdution

**Figure 1:**
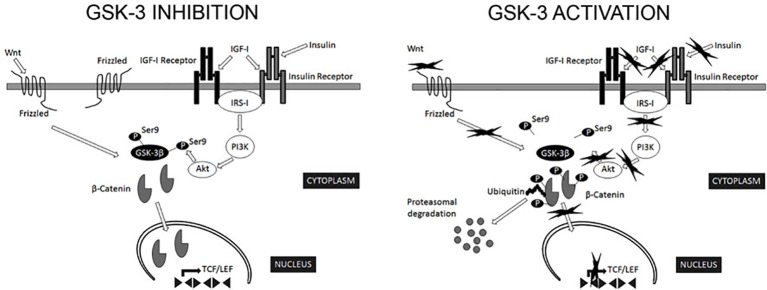
Regulation of GSK-3β activity. GSK-3β is constitutively active in most tissues and most commonly regulated by inhibitory phosphorylation on Ser9. The most relevant extracellular signaling pathways known to negatively regulate GSK-3β activity are those of insulin/insulin-like growth factor I and Wnt. In the canonical Wnt signaling pathway, Wnt stabilizes levels of β-catenin. Subsequently, stabilized β-catenin initiates the transcription of target genes. GSK-3β phosphorylates several components of this transduction pathway, β-catenin being the most widely characterized. Phosphorylated β-catenin is recognized by ubiquitin and targeted for proteasomal degradation (Wu and He, 2006). In addition, Akt phosphorylate Ser9 of GSK-3β in the context of insulin signaling pathway (Sutherland et al., 1993). Consequently, signals that modify GSK-3β activity are expected to alter β-catenin levels (Forde and Dale, 2007). From Llorens-Marítin et al. (2014), CC License.

Serine/threonine kinase glycogen synthase kinase-3 (GSK-3) is an essential human kinase, which is able to interact with more than 100 known substrates (Beurel et al., 2015) [see Figure 1 detailing the mechanisms of activation/inhibition (Llorens-Marítin et al., 2014)]. Starting with the 1990s, GSK-3 received increased attention due to two discoveries. First, it was shown to be essential to the abnormal phosphorylation of the tau protein, the process believed to cause neurofibrillary tangles in Alzheimer’s disease (AD) (Hanger et al., 1992); thus, causing a surge in studies trying to identify the potential uses of pharmacological agents for the treatment of this disease (Beurel et al., 2015). GSK-3 seems to be pivotal to the development of AD through tau hyperphosphorylation, memory impairment, increased production of beta-amyloid, increased inflammatory response, decreased acetylcholine synthesis, apoptosis (Wang et al., 2009), altered axonal transport (Hooper et al., 2008), or decreased synaptic plasticity (neuronal polarity). Because of these effects, various researchers have tried to test whether GSK-3 inhibition can be useful for the treatment of AD (del Ser, 2010; del Ser et al., 2013). Currently, there are few phase-II (A and B) clinical trials evaluating the potential usefulness of Tideglusib in Alzheimer’s disease (del Ser, 2010; del Ser et al., 2013), although the results are still inconclusive. The second event that led to the popularity of GSK-3 was the discovery of the inhibition of GSK-3 by lithium (Klein and Melton, 1996), a known mood-stabilizer. This association leads to the detection of other targets for GSK-3, which could be used in various psychiatric disorders (Beurel et al., 2015).

GSK-3 has been studied as a potential therapeutic target in many disorders, including neuropsychiatric [bipolar disorder (Klein and Melton, 1996), depression (Jope, 2011), anxiety (Sachs et al., 2013), and schizophrenia (Singh, 2013)], neurological [Alzheimer’s disease, supranuclear palsy (Tolosa et al., 2014), fragile X syndrome (Franklin et al., 2014), multiple sclerosis (De Sarno et al., 2008), Parkinson’s disease (King et al., 2001), Huntington’s disease (Scheuing et al., 2014), stroke (Chuang et al., 2011), traumatic brain injury (Leeds et al., 2014), and spinocerebellar ataxia type 1 (Leeds et al., 2014)], inflammatory diseases [sepsis (Hu et al., 2006), asthma (Bao et al., 2007), arthritis (Cuzzocrea et al., 2006), colitis (Whittle et al., 2006), and peritonitis (Jope et al., 2007)], or various types of cancers. The most studied associations between GSK-3 and human diseases involved either Alzheimer’s disease or neoplastic disorders.

There are few classes of GSK-inhibitors, including lithium (Martinez et al., 2011), the small peptide L803mts^10^, and members of the thiazolidinedione family, containing non-competitive inhibitors of GSK-3, such as TDZD-8 (Shapira et al., 2007) or Tideglusib^®^ (Noscira, Madrid, and Spain), the latter having an irreversible inhibitory effect on GSK-3 (del Ser et al., 2013). The inhibition of the GSK-3 pathways through distinct mechanisms has been associated with a wide range of adverse reactions, ranging from mild, such as vertigo—or diarrhea (del Ser et al., 2013)—to very severe, such as hypoglycemia—or tumorigenesis (Martinez et al., 2011). The use of Tideglusib specifically was associated with mild-moderate adverse reactions, which included transient increases in serum creatine kinase, ALT—or gGT—diarrhea, nausea, cough, fatigue, and headache (del Ser et al., 2013). In a phase-IIa clinical trial, the treatment was discontinued in 35% of all the active subjects, mainly due to adverse reactions (del Ser et al., 2013).

A significant role of GSK-3 is represented by its capacity to modulate human stem cells *in vivo*. Sato et al. showed that GSK-3 inhibition through 6-bromoindirubin-3′-oxime activates the Wnt pathway, maintains an undifferentiated phenotype of embryonic stem cells, and retains the pluripotent state-specific transcription factors Rex-1, Oct-3/4, and Nanog (Sato et al., 2004). Based on this effect, a recent study by Neves et al. evaluated the potential for natural tooth repair by using small molecule GSK-3 antagonists, such as Tideglusib. In their study, they used adult CD1 wild-type mouse first molars, damaged through controlled drilling. The holes were filled in with Pro-root mineral trioxide aggregate or Kolspon alone or in association with 5 μM CHIR99021 (SIGMA), 50 nM BIO (SIGMA), or 50 nM Tideglusib (SIGMA), and sealed with 3 M Ketac-Cem Radiopaque. Micro-computed tomography scanning showed increased mineralization with all the three substances; the increase was statistically significant at 4 and 6 weeks compared to the baseline, but there were no statistically significant differences between 4 and 6 weeks (Neves et al., 2017). The drug seems to act by activating the Wnt/β-cat signaling pathway of resident mesenchymal stem cells from the tooth pulp. The result, on condition of replicability on human subjects, may lead to a pharmacological treatment for cavities (Neves et al., 2017). However, before potentially using such therapies for dental cavities, a closer look should be taken at some of the main ethical issues posed by the use of this drug, and the main potential problems arising from their indiscriminate usage should be emphasized.

## Respect for Autonomy

Respecting the autonomy of the patient is one of the cornerstones of the contemporary bioethics, and is, as a general rule, generated through the process of obtaining the informed consent from the patients who are able to receive proper, relevant medical information, understand it, and make decisions based on their internal convictions rather than external influences (Faden Ruth and Beauchamp, 1986; Morar et al., 2008; Hostiuc, 2014).

A potential treatment for cavities with Tideglusib (or other GSK-3 molecules) is in its early stages, as the results have only been confirmed on mice; however, the extrapolation of the results to human subjects should be done only after a proper efficacy on animal models has been completely established. Furthermore, the conversion of medical research to clinical practice should be carried out only after an appropriate analysis of the potential side effects has been performed, which should include an elaboration of the long-term consequences. In recent years, there have been studies suggesting a potential tumorigenic effect of GSK-3 inhibition (Ding et al., 2007; Li et al., 2009; Ko et al., 2016; Zhang et al., 2016). This effect, however, has been largely refuted (Li et al., 2009; Wada, 2009; Zhou et al., 2012; Kunnimalaiyaan et al., 2015; Thorne et al., 2015; Chen et al., 2016; Nishimura et al., 2016), because the studies reporting such an effect have a small number of subjects to whom drugs had been systemically administered. It needs to be noted that an intra-dental application might lead to an increased release time of the drug, potentially causing significant local effects. Therefore, before allowing the usage of this drug, which alters numerous critical regulatory pathways, larger scale studies, should be performed to assess correctly the risk profile associated with its use; in turn, prospective patients should be properly informed to be able to take a genuinely informed decision, without which patients will not have enough relevant data for such a decision.

The understanding of information can be influenced by the fact that most studies carried out on this molecule have been conducted by highly specialized teams, and that the published results are not readily understandable by clinicians (either from clinical medicine or dentistry). These are difficult to understand phrases by average physicians’ and often need a “translation” from the highly specific medical jargon to an “average” one to be properly comprehended. This translation can be incorrectly done by either the physicians who try to understand its meaning and potential clinical significance, generating important errors by misinterpreting the results, or by other researchers who often oversimplify the results to reach a wider audience. If the information is altered “in translation,” its transmission toward patients could be erroneous, invalidating the obtained consent.

Another major factor that could alter significantly the understanding of relevant information is the mass media. For example, in an article published by the *Telegraph*, the authors stated that, “Tideglusib has already been shown to be safe in clinical trials of patients with Alzheimer’s disease so scientists say that the treatment could be fast-tracked into dental practices” (Knapton, 2017). The safety of the drug has not been properly tested, as it was only assessed on a small-scale, phase-II clinical trials on subjects who had very strict inclusion and exclusion criteria. Therefore, the information, as presented in the abovementioned article, is obviously incomplete and potentially incorrect.

When the medical information is transmitted to patients, there is an additional loss of meaning, as they often do not understand everything physicians tell them. This transfer is mainly altered by the use of unclarified medical jargon (Castro et al., 2007), which many physicians are often over confident in the capacity of their patients to understand the relevant information. For example, Castro et al. conducted a study in which they tried to describe the use of jargon by physicians for patients with diabetes and limited health literacy. The researchers audiotaped 74 outpatient encounters and coded the unclarified jargon by assigning each a clinical function. In 81% of all the encounters, there was at least one unclarified jargon term, and the mean occurrence of jargon terms was four per visit (Castro et al., 2007). Physicians can use medical jargon intentionally to hide “hurtful truths” or their lack of knowledge or unintentionally by being overconfident in the capacity of their patients for understanding them (Chapman et al., 2003; Abbe et al., 2006; Snow et al., 2007; Deuster et al., 2008; Williams et al., 2008). The same is true in dentistry. For example, Mortensen et al. showed a low-rate recall by children and their parents in orthodontic treatments regarding the risks (relapse, caries, and periodontal problems), reasons for treatment, procedures, and responsibilities of the children during treatment (Mortensen et al., 2003).

## Transfer of Data Between Researchers

**Figure 2:**
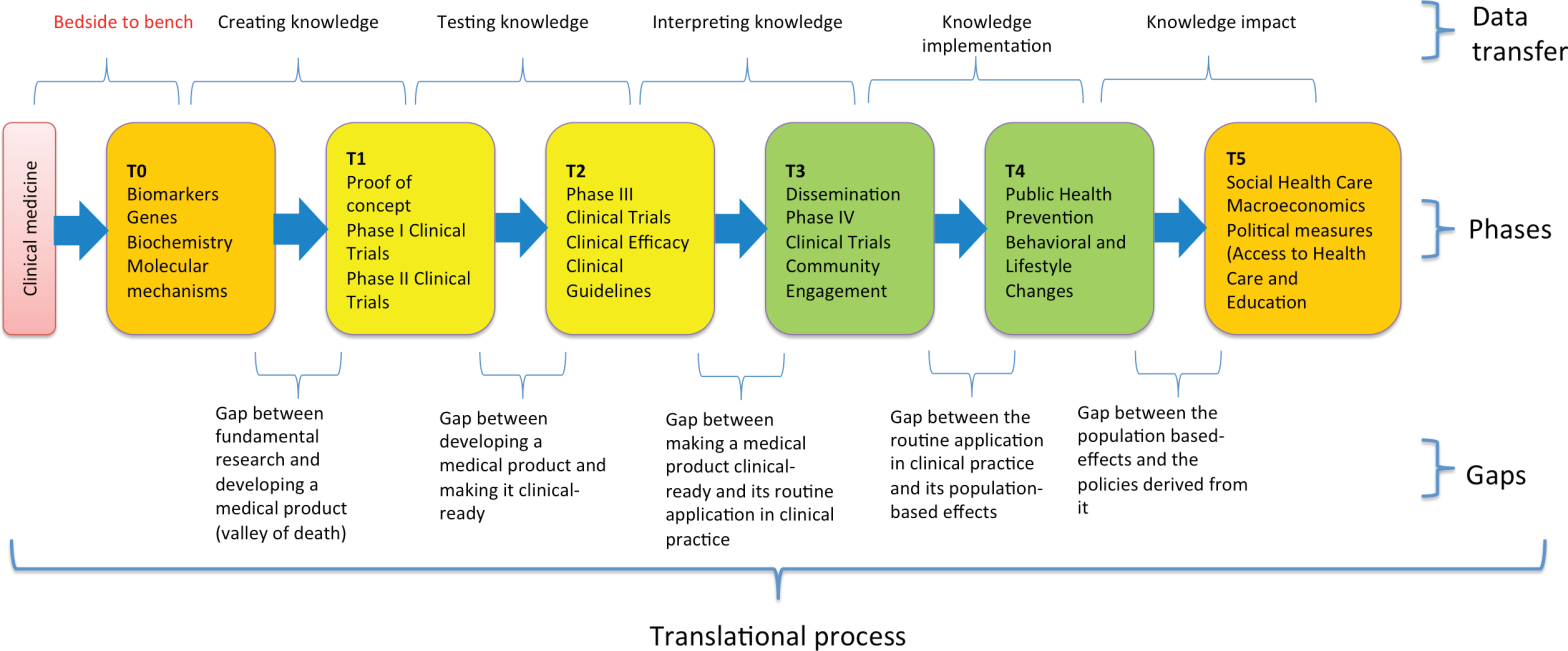
The translational research process, from fundamental research to developing policies (Hostiuc et al., 2016), CC License.

The potential usage of Tideglusib for dental repair can be seen as a typical example of translational/interdisciplinary research (Figure 2) (Hostiuc et al., 2016), in which fundamental information is translated both vertically (to develop therapeutic effects that were envisaged from the early, preclinical stages) and horizontally toward other unexpected therapeutic effects. When transferring information from bench to bedside, there is a decrease in the quantity of the retained data about the detailed, biomolecular mechanisms of action of the particular drug, and an increase in the number of the investigators, who are using the data and applying them to clinical environments. Therefore, in later phases of clinical trials, we have a high number of investigators, assistants, and technicians, who lack a complete understanding of the effects of a specific drug (i.e., Tideglusib), affecting biochemical/molecular pathways and their connections with the other pathways. When doing a horizontal translation, focusing on a different effect than the one initially envisaged, investigators use the data that often contains lesser information about the intrinsic biomolecular mechanisms, staying at the base of the new therapeutic effect. In this case, medical/dental investigators most likely will have a decreased understanding of the data compared to the investigators in the neurosciences field. If a proper analysis of the molecular pathways and interactions is not performed, there is an increased risk of generating significantly late adverse consequences. The typical example of this horizontal translation, with substantial negative effects, is Thalidomide. Initially developed as an anticonvulsant, it only showed modest positive effects; however, soon it was revealed that it also induced a deep sleep with no hangover, which caused it to be used as a hypnotic, sedative, and tranquilizer. Moreover, it was shown to be useful for the treatment of the morning sickness in pregnant women and began to be widely used for this purpose, without a proper research of the potential negative effects, and despite the fact that afterward some investigators showed that it caused fetal deformities among animal models (Lenz, 1988; Randall, 1990). Therefore, before a potential usage of Tideglusib for dental cavities is tested in clinical practice, investigators should go again “to the bench” and reevaluate the inherent risks in association with the new therapeutic purpose, especially taking into account the difficulties posed by adequately understanding highly complex, biomolecular information by clinicians.

## Dental Beneficence Versus General Non-Maleficence

Two fundamental ethical principles that collide over the potential use of Tideglusib (or other GSK-3 inhibitors) for dentine regeneration are beneficence and non-maleficence. According to the principle of beneficence, physicians are morally obliged to act for the benefit of their patients. According to Beauchamp and Childress, beneficence includes five main types of actions: 1) protect and defend the rights of others, 2) prevent harm that may occur to others, 3) remove conditions that could cause harm to others, 4) help persons with disabilities, and 5) rescue persons in danger (Beauchamp and Childress, 2005). According to the principle of non-maleficence, physicians are obliged not to harm their patients in any way. The primary distinction between beneficence and non-maleficence lies in the action that causes the effect. If in beneficence, physicians are required to act either to do good or to remove harm, in non-maleficence, physicians must not take any action that could harm their patients.

Using Tideglusib for the treatment of dental cavities is a positive action, directed toward removing a condition that can cause harm; therefore, recommending and following this procedure (if shown useful by follow-up studies) could generate beneficence. However, Tideglusib may have short- or long-term, local or general consequences, which have to be taken into account before recommending it. For example, phase-II clinical trials showed that Tideglusib temporarily increases transaminase levels, which reach values a few times higher than normal. However, there is a large population of patients, who suffer from liver disorders, either clinical or subclinical, which could be decompensated by the usage of such a drug, potentially causing life-threatening events, such as portal encephalopathy or acute liver failure. A clinical study aimed at analyzing the effects of Tideglusib on patients with liver disorders and taking into account the increase in transaminase levels in the initial patients would be morally problematic too, as it may simply breach the principle of non-maleficence.

Another potential issue about using this treatment on humans is the fear of adverse reactions associated with the systemic distribution of the drug. However, in the abovementioned study (Neves et al., 2017), the doses needed for tooth repair were around 1,000 times lower, compared to those used in the clinical trials for neurological disorders (Neves et al., 2017). Lithium, another GSK-3 inhibitor, has been shown to have mild teratogenic effects in humans, potentially causing cardiac malformations, such as Ebstein anomaly or increased birth weight (Giles and Bannigan, 2006) through an incompletely described mechanism. To our knowledge, the teratogenic effects of Tideglusib have not been evaluated. Therefore, it is not yet known whether they are safe for use in pregnancy, and if not, which doses pose the most danger; even very small doses could have important effects on the embryo/fetus.

When two ethical principles collide, only one should be acted upon, with the other being infringed (the weaker one in that particular case). Regarding the potential use of Tideglusib for the treatment of dental cavities, beneficence is the weaker principle. As shown in the published studies, the risks seem minor, and the scale of studies assessing them was small and failed to include a wide array of subjects with various pathologies or physiological conditions (e.g., pregnancy). Moreover, as noted above, GSK-3 is involved in more than 100 biochemical pathways and potentially in dozens of diseases. Until these risks are properly evaluated and the physiology of GSK-3 and its role in pathogenesis is well understood, we argue *against* the translation of this drug in dentistry. At least at this stage, avoiding harm should be more important than doing good. Therefore, a fast track of the drug to clinical dentistry should not be allowed, as it may cause more harm than good.

## Conclusion

The potential usage of Tideglusib for dental cavities should be tested thoroughly in clinical practice. It should respect the same strict ethical and regulatory criteria from clinical medicine. A translation of the potential risks should be done only after large-scale, phase-III/IV clinical trials, explicitly designed to test the usefulness of this drug in dental medicine.

## Author Contributions

All authors participated equally in the developing of the idea, drafting the manuscript, correcting it and approving the final version.

### Conflict of Interest Statement

The authors declare that the research was conducted in the absence of any commercial or financial relationships that could be construed as a potential conflict of interest.
